# Incident Factors in the Sustainable Development of Digital Teaching Competence in Dual Vocational Education and Training Teachers

**DOI:** 10.3390/ejihpe11030054

**Published:** 2021-07-15

**Authors:** Jesús Sánchez-Prieto, Juan Manuel Trujillo-Torres, Melchor Gómez-García, Gerardo Gómez-García

**Affiliations:** 1 Department of Didactics and Theory of Education, Autonomous University of Madrid, 28049 Madrid, Spain; jesus.sanchezprieto@estudiante.uam.es (J.S.-P.); melchor.gomez@uam.es (M.G.-G.); 2 Department of Didactics and School Organization, University of Granada, 18071 Granada, Spain; jttorres@ugr.es

**Keywords:** digital competence, ICT competences, teacher education, influential factors, dual training

## Abstract

For years the technological revolution has been transforming all facets of our society. Teaching could obviously not be an exception but is quite the opposite because of its role in training the individuals of that society. Teachers at all levels of education are subjected to an adaptation process to develop the digital skills necessary for this transformation. This process must be permanent as there are still major deficiencies in teachers’ ICT knowledge and rejection of their application. This study aims to see whether the inadequacy of digital teaching skills also occurs in the Dual Vocational Training modality. To this end, a descriptive quantitative method has been carried out in a sample of teachers from the Professional Formation Dual system in the Autonomous Community of Andalusia. The results show an insufficient level of digital skills that is therefore improvable, finding some factors that influence, to some extent, the various components of digital competence such as prior teacher training, the locality in which their school resides or the category of teaching to which he belongs. Therefore, it is advised to continue to promote in-service training in digital competence for in-service teachers in order to achieve sustainable educational development.

## 1. Introduction

ICTs (Information and Communication Technologies) have transformed social relations in recent decades in all its aspects: communication, coexistence, work, education, etc. [1]. Nowadays, the higher the level of ICT knowledge is, the greater the chances of easily achieving quality and higher-paid jobs [2]. ICT in education offers great advantages such as increasing student motivation, growing learning resources, improving knowledge understanding and attention to diversity, among others [3,4].

However, the use of new technologies in themselves does not improve the learning teaching process. The teaching methodology needs to change in order to benefit from ICT, not only by using ICT as a resource but as a way of learning. It is therefore about changing the curriculum of teachers and the methodologies used [5,6].

The teacher is the main protagonist in this techno-pedagogic transformation. He is in charge of carrying out new functions and tasks, since he not only transmits information, but his fundamental role is to facilitate the learning of students by designing new learning environments and carrying out the role of tutor, counselor and moderator, so having good teaching practices with Information and Communications Technologies (ICT) is essential [7].

Although logically teachers need to have quality digital-pedagogical training in or-der to make this change, launching educational research programs can make the existing resources useful [8]. Teachers should be adequately trained to integrate the use of ICTs in the classroom.

### 1.1. Digital Competences in Teaching, Origin and Standards

The concept of digital competence has its origin in a new vision of learning in formal studies that starts from the need to classify those skills and aptitudes that the individual must acquire and consolidate as an essential means to advance in his or her academic career and, subsequently, throughout life [9].

The European Commission [10] understands digital competence as the safe and critical use of ICT in the Information Society for work, leisure and communication. It is based on basic ICT skills: the use of computers to obtain, evaluate, store, produce, present and exchange information and to communicate and participate in collaborative networks (p.15). If we look specifically at the concept of digital teaching competence, according to Flores and Roig [11], it is a type of multidimensional competence and can be defined as the ability to mobilize those skills and abilities that allow you to search and critically select, obtain and process relevant information using ICT to transform it into knowledge, while being able to communicate such information through the use of different technological and digital media, acting responsibly, respecting the socially established rules and taking advantage of these tools to inform, learn, solve problems and communicate in different scenarios of interaction.

The National Institute of Educational Technologies and Teacher Training (INTEF) defined digital competence as “the creative, critical and secure use of information and communication technologies to achieve the objectives related to work, employ-ability, learning, free time, inclusion and participation in society”.

The Common Framework for Digital Teaching Competence was developed by the INTEF and is considered a good tool for planning and evaluating training actions for teachers. According to him, digital skills are classified into five airlines [12] (Table 1):

### 1.2. Factors Influencing Teacher’s Digital Competence

In recent years, certain factors have been investigated that condition the level of digital skills of teachers, among which the age and gender of the teacher have been the most studied.

However, not as much research has been done on other factors that may affect these teaching digital competencies, such as:

(a) The “previous teacher training”, which positively influences the digital competencies that they possess as suggested by Pozo et al. [13] in their “Correlal analysis of the incident factors in the level of digital competence of teachers” highlighting the importance of continuous training and the special correlation in the event that the teacher’s training is technological. However, there are also inputs indicating that the number of training courses does not imply a higher level of teaching [14].
(b) The “teaching experience” is another factor also studied finding discrepancies in the revision of literature. Authors such as Muñoz and González [15] or Ortiz [16] note that, although sometimes experience positively influences the level of digital teaching competence, in other cases, the relationship is negative as teachers do not adapt to new teaching systems, mainly older ones, resisting changes.

Zempoalteca, González, Barragán and Guzmán [17] reveal that new-income teachers suffer from digital competence, but with teaching experience, they are improving in this regard, which justifies the importance of lifelong learning to motivate the use of ICT. Within the areas of digital competences it emphasizes that the one that influences the most teaching experience is the “digital security level” according to Pozo et al. [13].

(c) The third factor to consider and perhaps the one that has been studied the most in recent years is that of the influence of the “educational stage or level of studies” where the teacher is located. Authors such as [18,19,20] indicate that the higher the degree of studies of teachers the digital teaching competence improves in their teaching practice.

By areas of competence, Pozo et al. [13] detect that high school teachers have higher values than primary schools in terms of digital literacy, but that primary schools have greater communication and collaboration capacity and those of early childhood education excel in the creation of content. However, there have also been jobs where digital methodological change is found to be greater in early childhood and primary education than in high school [18,19,20,21].

As for the stage of Vocational Training, which is what we are dealing with in this article, few studies have been found, although Fernandez de la Iglesia [22] provides that vocational training teachers stand out about the other formative stages in the level of digital skills.

(d) On the other hand, the “professional family of the teacher” is a variable that can also influence teachers. Pozo et al. [13] detects the greatest digital skills in teachers in technology related areas. In this way, Echegaray [18] highlighted the disciplines of Computer Science and Mathematics as the most qualified.

However, a Falcó study [23] detected Physical Education and surprisingly Mathematics as the areas that valued ICT the least. It is also of note that Muñoz and González [15] and Zubieta, Bautista and Quijano [24] found that teachers in administrative science areas used very little digital skills in their teaching practice.

(e) Finally, in literature, other factors have been found that influence the level of digital competence of the teacher by factors of a more subjective nature. Kounenou, Roussosb, Yotsidic and Tountopoulouc [25] and Gil, Rodríguez and Torres [26] allude to the attitudes of teachers, their assessment of ICT, the interests that move them or their liking to them as decisive factors in the level of digital competences. The perceived need for digital training is perhaps the most important variable for the use of ICTs in the classroom. They also indicate that it also influences the teacher’s constructive approach to the teaching–learning process and a culture of collaboration.

In short, knowledge of the factors that condition the acquisition of digital teaching competence is crucial to establish methodologies and promote effective plans of continuous training of teachers in schools, considering the differences that exist between these factors.

### 1.3. Special Needs of Digital Skills in Dual Vocational Training

Vocational Training has played an essential role in training policies. In Professional Formation centres there has been technological transfer between the business world and schools, so Professional Formation must be bilateral for the resources to be optimized and for society to be more competitive and inclusive [27]. Professional Formation teachers have experienced the most change in the last 25 years, so they are used to facing changes in his practices in order to adapt to dual training, as they have done in the past with other transformations.

“Dual Vocational Training” means the mode of teaching that is carried out simultaneously in two different places: a training centre and a work centre of a company. Its purpose is to complement theoretical training and practice. In this modality, the training process and the evaluation process are carried out by both the teaching centre and the work centre.

One of the principles of dual training is for companies to participate in the design of the training project. According to some studies [28], less than a quarter of companies have participated in the curriculum or have let the most, which does not correspond to the definition of dual training.

The “collaboration” between the teaching centre and the work centre oriented toward competitive development in the digital field of the teacher framed in a process of innovation and change generated by an adequate use of ICT is a key element for the success of dual training, where two educational realities that work at very different rates and times are put in touch [29,30].

According to Rivera [27], dual quality training requires the following:

(a) Good compliance oversight to ensure that the training provided in the company is optimal.
(b) The implementation of a rigorous student evaluation process and creating a network of collaboration between schools and companies.
(c) Improving tutoring in both study centres and workplaces makes it essential to improve career and academic guidance through specific training courses, including digital competence.

Improving the digital skills of teachers at both the school and the workplace is essential. In the present context in which we find ourselves, in which digital transformation is leading most of the changes within the different present and future jobs, it is necessary to train the future professionals who make up this educational stage in digital competence in order to be able to face the current and future demands of the future labour context. [30].

On the other hand, it can be said that ICTs become more necessary in the dual model, since having a lesser presence of students in the classroom makes it necessary for tutors to use new technologies to track students [31].

As the number of students in Dual Professional Formation is increasing in recent years, and therefore the increase in teachers in this modality of teaching, coupled with the scarcity of research related to the subject, it is anticipated that this research will help present and future teachers to better understand what the elements of a methodology based on the use of information and communication technologies are.

### 1.4. Research objectives

The purpose of this study is to be able to establish a description of the reality analysed through descriptive and inferential statistics on the level of digital teaching competence presented by the faculty of Dual Education of the Autonomous Community of Andalusia (Spain). As a result of this main objective, in order to guide research, the following specific objectives are formulated:

- O1: Find out the level of digital teacher competence self-perceived by teachers of the Dual Vocational Training stage.
- O2: Analyse which sociodemographic factors can be considered as predictors of each dependent variable analysed and check their level of importance.

## 2. Materials and Methods

This work proposes the use of a descriptive quantitative methodological approach. The purpose is to be able to describe by means of statistical tests the educational reality concerning the level of digital competence in Dual Education teachers in Andalusia.

### 2.1. Sample

The study sample consists of a total of 1568 teachers from the dual vocational training stage of the Autonomous Community of Andalusia. The sampling chosen to select participants was by convenience. The questionnaire was sent to all teachers who teach at this stage of education in Andalusia (Almería, Granada, Jaén, Malaga, Córdoba, Sevilla, Cádiz and Huelva). The subjects were part of secondary education centres that offer the dual vocational training modality. Some of its main features are:

- Minimum age (18)
- Average age (33)
- Maximum age (49)
- Men (823)
- Women (745)

### 2.2. Instrument

With regard to the design of the instrument, an ad hoc questionnaire was set up on the basis of the five areas of digital competence proposed by the European Digital Skills Framework for Citizens (DigComp) [32]. In sum, a sixth related area was added to the technology tool domain.

Therefore, the dimensions covered by the questionnaire are defined as follows:

- Instructional Design and Learning (IDL).
- Communication and Collaboration of Digital Resources (CCDR).
- Creation of Digital Content (CDC).
- Digital Security Knowledge (CS).
- Digital Tools (DT).
- Problem Solving (PS).

In addition, different sociodemographic variables of interest were taken into account. Firstly, the participant’s gender (Gender), whether he/she had any previous ICT-related training (ICT.F), previous level of education (L.Stud), the teacher’s professional category (P.Categ), the professional family from which the teacher comes (P.Fam) and the number of inhabitants living in the town where he/she works as a teacher (Inhab). The following is a summary of these variables, as well as the different response options offered by the questionnaire in relation to these variables:

- Gender (Man = 0; Woman = 1).
- Previous ICT Formation (ICT.F) (Yes = 1; No = 0).
- Previous study level (L.Stud) (Degree = 0; Degree, Grade, Master = 1; Degree, Master = 2; PhD = 3; Professional Formation = 4; Grade = 5; Grade, Professional Formation = 6; Grade, Master = 7; Engineering = 8; Bachelor = 9; Bachelor, Master = 10; Master = 11).
- Professional Category (P.Categ) (Freelance = 0; Civil Servant =1; Intern = 2; Workers = 3).
- Professional family (P.Fam.) (Others = 0; Administration and management=1; Commerce and Marketing = 2; Languages = 3).
- Number of inhabitants of the population where he/she works (Inhab) (Between 100,000 and 1,000,000 = 0; Between 25,000 and 100,000 = 1; More than 1,000,000 = 2; Between 1000 and 5000 = 3; Between 5000 and 25,000 = 4; Less than 1000 = 5).

For the validation procedure, the instrument was subjected to a content analysis through multiple experts from different higher institutions (University of Seville, Malaga and Granada). Subsequently, its internal consistency of the variables was corroborated through the Barlett sphericity test (KMO .841; *p* < 0.001), which allowed us to move forward with the validation method. To this end, a factorial analysis by component analysis was used through the rotation technique known as the Varimax.2.3. Procedure

Rstudio statistical software was used in version 3.6.1 for data analysis. First, the common descriptive statistics were applied, which allowed us to approach the description of the perceptions of dual vocational training teachers. Subsequently, the outliers and the lost values were analyzed, the latter being omitted from the data frame. Two outliers were found, one for the variable age (55) and one for experience (25). In both cases, a manual function was defined with an R software setting that if the value of each of the variables was greater than the quantile or 95th percentile, it would be allocated by the median, while it is more robust than the average.

The feature engineering was then carried out. The original dataset was divided into two large sets: training and testing using the R initial split function with a prop of 85%. This means that the training dataset maintained 85% of the original data. This is because the training group was used to model the algorithm and the testing algorithm to evaluate its performance.

The independent metric variables, age and experience, were focused and applied to the transformation of Yeo-Johnson [33], which is an extension of the Box–Cox transformation. The categorical variables then became indicator variables or dummies. Finally, the scores of the different variables dependent on the study were added.

“Parsnip”, a new R package, was used for statistical analyses. The testing dataset was used to implement predictions. From these values, the root of the mean square error was calculated (RMSE).

## 3. Results

According to the results set out in Table 2, the level of digital teacher competence presented by teachers at the Dual Vocational Training stage can be considered as an average. The results obtained in all dimensions have been very similar but are below the level that is considered optimal. The level of variability is low so we can consider the low existence of outliers.

Consequently, the multiple linear regression models of each of the dimensions of the covered teaching digital competence were configured. Only the incident factors that were found to be statistically significant will be presented. They are defined as follows:

### 3.1. Instructional Design and Learning

The coefficients for intercept, digital tools, digital security, instructional design and learning and the number of inhabitants (Between 1000 and 5000 and less than 1000) were significant (Table 3). By comparing the linear regression model with a regression tree and an xgboost, it was obtained that the best model, in terms of root of the mean square error, was linear (RMSE = 4.02).

### 3.2. Communication and Collaboration of Digital Resources

The coefficients for intercept, digital tools, digital security, instructional design and learning and the professional category of the teacher (intern and workers) were significant (Table 4). By comparing the linear regression model with a regression tree and an xgboost, it was obtained that the best model, in terms of root of the mean square error, was linear (RMSE = 4.02).

### 3.3. Creation of Digital Content

The intercept was significant instructional desing and learning, digital tools, digital security, and the professional category of the teacher (intern and workers) were significant (Table 5). By comparing the linear regression model with a regression tree and an xgboost, it was obtained that the best model, in terms of root of the mean square error, was the linear (RMSE = 4.34).

### 3.4. Digital Security Knowledge

The intercept was significant, as was the creation of digital content, digital security, communication and collaboration of digital resources, problem solving and the previous study level of the teacher (in this case degree, grade and master) (Table 6). Comparatively, the xgboost was the best model to have a root of the mean square error (RMSE = 4.07) smaller than the linear (RMSE = 4.17).

### 3.5. Digital Tools

The intercept, creation of digital content, problem solving, digital tools, creation of digital content and previous ICT training were significant (Table 7). Comparatively, the xgboost was the best model to have a root of the mean square error (3.87) smaller than the linear (4.42).

### 3.6. Problem Solving

The intercept, digital security, instructional design and learning, professional category of the teacher (intern), previous ICT formation and digital tools were significant (Table 8). Comparatively, the linear regression model was the best model to have a root of the mean square error (RMSE = 3.84) smaller than the linear one (RMSE = 4.02).

## 4. Discussion

ICTs provide teachers with relevant benefits such as student motivation, better assimilation of knowledge or attention to diversity. This is why teachers’ mastery of different digital skills has become a challenge to address throughout the educational community in recent years.

The teacher performs the main task in this transformation of teaching since he is the one who must launch the new practices based on ICT, which are not limited to the transmission of information but also aide in being an advisor and facilitator of the learning of his students, simultaneously performing the functions of tutor, moderator and counselor. In particular, the immersion of these resources in vocational education, such as that at hand, is considered essential, as this is a stage in which it is necessary to convey to future generations of professionals the importance that lies in digital transformation in the face of the future challenges facing the labour society [6,34,35].

In this way, the main objective of this article was to show the level of self-perceived digital competence that teachers have of the educational stage of dual vocational training, as well as to indicate whether sociodemographic factors influence the development of these perceptions.

The results obtained by the study indicated that the level of digital skills of dual vocational training teachers can be considered similar to that of other educational stages [24,36,37], also being below what would be necessary for optimal teaching use of ICT resources. We are still in a state-of-the-art phase that shows self-perceived levels that are in the average based on low data from teachers, which could indicate that teachers are not yet able to integrate ICTs into the classroom or do not have the necessary training to make optimal use of them.

In view of the analysis of the sociodemographic factors affecting the development of these competencies, the results of this work reflected that, first, the number of inhabitants of the population where teaching was performed was a component that affected the level of communication and digital collaboration between the different teachers of this stage. Specifically, in municipalities whose population was small, there are higher levels of collaboration between the different teaching professionals since the nodal networks are narrower.

In this regard, it was also revealed that the previous level of studies was an incident in presenting a greater self-perception of the level of digital security presented by the teachers analyzed. It continues in the same line as previous studies [13] that ensure that previous training is a determining factor in the development of some of the digital competencies. However, as opposed to other works [20], this work also shows that not having a higher level of academic study leads to greater digital prowess, as those population sets with greater academic prowess (L.Stud 3; Lstud 6) did not have significance in the model configuration, unlike the set of teachers that showed dependency relationship as DS (Lstud2).

In the same line, the previous ICT training presented by some teachers who participated in this study was configured as another determining factor in establishing concrete knowledge about digital tools, as well as in problem solving. In this sense, it is clear to elucidate a relationship between prior digital training and knowledge about digital resources. The knowledge of technological resources also promotes a better resolute ability to deal with a problematic situation of this nature.

Finally, the category of teacher was served as an incident factor, which showed significance in problem solving, in which all the official professors showed significant differences in their responses. On the other hand, when creating digital content, the set configured by interim teachers established an incident factor in the development or not of this construct. In this sense, it is followed in a line similar to Muñoz and González [15] or Echeverría [18] in which they indicate the possibility of a better adaptation to technological immersion by young teachers or that he still has a permanent job.

In short, different sociodemographic variables are presented as determinants in being able to indicate a greater or lesser self-perception about the level of digital competence in dual vocational training teachers, who, as shown by the results of this work, rate their digital competence level as scarce.

Therefore, this research work serves to establish an awareness-raising exercise within the teaching profession. It is necessary to continue to promote ongoing digital training for teachers in view of the many current educational challenges that require this digital transformation process. This work highlights this need within the dual vocational training stage, which also aims to train future professionals who will cover the labour landscape of the near future. In short, there is a need for updated teachers with optimal digital skills to provide an adequate training service to these students.

## 5. Conclusions

There is no doubt about the importance of teachers’ digital knowledge today, as the application of new technologies in teaching leads to teaching models where communication makes teaching more flexible, collaborative, relocated, individualized and dynamic.

For ICTs to be truly effective in teaching, teachers must have instrumental and pedagogical digital training, and for this, they need to have access to training programmes that make the available technological resources really useful. This will be achieved with quality training in digital skills in both initial teacher training and life-long learning.

In Dual Vocational Training, digital teaching skills take on special prominence as, in this model, collaboration between the school and the workplace is essential and improves significantly with the use of ICTs from teachers in both fields. In addition, the use of digital knowledge is essential in dual education to keep an optimal track of students as they have less face-to-face presence in classrooms than in other models.

With regard to the limitations of the study, the main one is that it only provides for the dual formation of a single autonomous community, so it is proposed as a future line of research to extend the research to the rest of Autonomous Communities of the national territory with the intention of comparing and checking whether the results are similar.

Therefore, digital teaching competence is still a challenge to be addressed by the different professionals that make up the education system. It is necessary that, in the face of the challenges of digital transformation that our current society experiences, from the educational scene, quality digital training is promoted permanently for the whole body of teachers.

## Figures and Tables

**Table 1:** Competency area. Source: [12].

**Area 1. Instructional Design and Learning**	Ability to identify, locate, obtain, store, organize and analyze digital information, data and digital content, evaluating its purpose and relevance to teaching tasks.
**Area 2. Communication and collaboration**	It consists of communicating in digital environments, sharing resources through online tools, connecting and collaborating with others through digital tools, interacting and participating in communities and networks; intercultural awareness.
**Area 3. Creation of the digital content**	Ability to create and edit new digital content, integrate and rework previous knowledge and content, make artistic productions, multimedia content and computer programming.
**Area 4. Security**	It refers to the protection of information and personal data, protection of digital identity, protection of digital content, security measures and responsible and secure use of technology.
**Area 5. Problem solving**	Solve conceptual problems through digital means, use technologies creatively, solve technical problems and update their own competence and other people’s competences.

**Table 2:** Results of types of competences.

Dimension	Mean	SD	Skew	Kurtosis
IDL	2.498	1.112	−0.231	−1.345
CCDR	2.493	1.091	0.345	−1.247
CDC	2.510	1.123	−0.044	−1.214
DT	2.502	1.132	0.034	−1.247
DS	2.503	1.124	0.027	−1.347
PS	2.486	1.007	0.348	−1.278

Note: IDL (Instructional Design and Learning); CCDR (Communication and Collaboration of Digital Resources); CDC (Creation of Digital Content); DT (Digital Tools); DS (Digital Security Knowledge)*;* PS (Problem Solving).

**Table 3:** Multiple linear regression model IDL.

Term	Estimate	Std.Error	Statistic	*p* Value
(Intercept)	16.38153	1.57906	10.37423	***
DT	0.09882	0.032099	3.078571	0.002135
DS	0.093998	0.031037	3.028598	0.002518
IDL	0.087089	0.031019	2.80757	0.005085
Inhab3	−1.02257	0.403622	−2.53348	0.01144
Inhab5	−0.86797	0.391592	−2.21651	0.026873

Note: DT: Digital Tools; DS: Digital Security; IDL: Instructional Design and Learning; Inhab: Number of inhabitant of the population where it is taught; ***: *p* value < 0.000001.

**Table 4:** Multiple linear regression model CCDR.

Term	Estimate	Std.Error	Statistic	*p* Value
(Intercept)	13.3167	1.523191	8.742635	***
DT	0.157718	0.030283	5.208103	***
IDL	0.141077	0.029296	4.81552	***
DS	0.123197	0.02941	4.189032	***
P.Categ2	−1.02537	0.324797	−3.15697	0.00164
P.Categ3	−0.69475	0.313281	−2.21767	0.026794

Note: DT: Digital Tools; IDL: Instructional Design and Learning; DS: Digital Security; P.Categ: Professional Category; ***: *p* value < 0.000001.

**Table 5:** Multiple linear regression model CDC.

Term	Estimate	Std.Error	Statistic	*p* Value
(Intercept)	11.9745	1.34659	7.94582	***
ILD	0.7891	0.09471	5.64541	***
DT	0.94823	0.04651	3.95845	***
DS	0.34897	0.31231	4.64823	***
P.Categ3	−1.02515	0.26458	−2.64846	***
P.Categ2	−0.8769	0.34564	−3.64826	0.00234

Note: IDL: Instructional Design and Learning; DT: Digital Tools; DS: Digital Security; P.Categ: Professional Category; ***: *p* value < 0.000001.

**Table 6:** Multiple linear regression model CS.

Term	Estimate	Std.Error	Statistic	*p* Value
(Intercept)	14.08923	1.538191	9.15961	***
CDC	0.16192	0.03109	5.208103	***
DS	0.101674	0.029884	3.402338	0.000694
CCDR	0.091824	0.029827	3.078571	0.002135
PS	0.07776	0.030701	2.532863	0.01146
L.Stud1	−1.16003	0.56476	−2.05402	0.040225

Note: CDC: Creation of Digital Content; DS: Digital Security; CCDR: Communication and Collaboration of Digital Resources; PS: Problem Solving; L.Stud: Previous study level; ***: *p* value < 0.000001.

**Table 7:** Multiple linear regression model DT.

Term	Estimate	Std.Error	Statistic	*p* Value
(Intercept)	12.85503	1.605328	8.007727	***
CDC	0.135327	0.032305	4.189032	***
PS	0.110781	0.031668	3.498226	0.000488
DT	0.108786	0.031974	3.402338	0.000694
CCDR	0.093453	0.030857	3.028598	0.002518
ICT.F1	0.546624	0.234687	2.329156	0.020043

Note: CDC: Creation of Digital Content; PS: Problem solving; DT: Digital Tools; CCDR: Communication and Collaboration of Digital Resources; ICT: Previous ICT Formation; ***: *p* value < 0.000001.

**Table 8:** Multiple linear regression model PS.

Term	Estimate	Std.Error	Statistic	*p* Value
(Intercept)	16.538	1.530233	10.8075	***
DS	0.105484	0.030153	3.498226	0.000488
IDL	0.086504	0.030176	2.866677	0.004232
P.Categ2	−0.92286	0.332532	−2.77525	0.005615
ICT.F1	0.615947	0.228807	2.691992	0.007217
DT	0.079221	0.031277	2.532863	0.01146

Note: DS: Digital Security; IDL: Instructional Design and Learning; P.Categ: Professional Category; ICT: Previous ICT Formation; DT: Digital Tools; ***: *p* value < 0.000001.
